# Tyrosine kinase inhibitors in patients with advanced anaplastic thyroid cancer: an effective analysis based on real-world retrospective studies

**DOI:** 10.3389/fendo.2024.1345203

**Published:** 2024-02-26

**Authors:** Bo-Hua Kuang, Wen-Xuan Zhang, Guo-He Lin, Chen Fu, Ru-Bo Cao, Bi-Cheng Wang

**Affiliations:** ^1^ Cancer Center, Union Hospital, Tongji Medical College, Huazhong University of Science and Technology, Wuhan, China; ^2^ Institute of Radiation Oncology, Union Hospital, Tongji Medical College, Huazhong University of Science and Technology, Wuhan, China; ^3^ Department of Oncology, The Second Affiliated Hospital of Anhui Medical University, Hefei, China; ^4^ Wuhan No.1 Hospital, Wuhan, China

**Keywords:** anaplastic thyroid cancer, tyrosine kinase inhibitor, response rates, survival outcomes, real-world retrospective analysis

## Abstract

**Background:**

Tyrosine kinase inhibitors (TKIs) contribute to the treatment of patients with anaplastic thyroid cancer (ATC). Although prospective clinical studies of TKIs exhibit limited efficacy, whether ATC patients benefit from TKI treatment in real-world clinical practice may enlighten future explorations. Therefore, we conducted this effective analysis based on real-world retrospective studies to illustrate the efficacy of TKI treatment in ATC patients.

**Methods:**

We systematically searched the online databases on September 03, 2023. Survival curves were collected and reconstructed to summarize the pooled curves. Responses were analyzed by using the “meta” package. The primary endpoints were progression-free survival (PFS), overall survival (OS), objective response rate (ORR), and disease control rate (DCR).

**Results:**

12 studies involving 227 patients were enrolled in the study. Therapeutic strategies included: anlotinib, lenvatinib, dabrafenib plus trametinib, vemurafenib, pembrolizumab plus dabrafenib and trametinib, pembrolizumab plus lenvatinib, pembrolizumab plus trametinib, and sorafenib. The pooled median OS and PFS were 6.37 months (95% CI 4.19-10.33) and 5.50 months (95% CI 2.17-12.03). The integrated ORR and DCR were 32% (95% CI 23%-41%) and 40% (95% CI 12%-74%).

**Conclusion:**

In real-world clinical practice, ATC patients could benefit from TKI therapy. In future studies, more basic experiments and clinical explorations are needed to enhance the effects of TKIs in the treatment of patients with ATC.

## Introduction

Most patients with anaplastic thyroid cancer (ATC) have an extremely poor prognosis and survive less than half a year from the disease diagnosis, with a 1-year overall survival (OS) of 20% (1, 2). Chemotherapy, surgery, and radiotherapy are the classic treatments for ATC. In recent years, targeted therapy and immunotherapy have brought novel survival benefits in patients with advanced ATC (3). However, significantly prolonging the survival time can be the biggest challenge.

Tyrosine kinase inhibitors (TKIs) have greatly improved the survival outcomes of multiple malignant tumors. In patients with radioiodine-refractory differentiated thyroid cancer, TKI therapy (lenvatinib) has been certificated to be associated with prolonged OS (HR 0.65, 95% CI 0.52-0.81) and progression-free survival (PFS) (HR 0.24, 95% CI 0.19-0.31) compared with placebo (4). Moreover, TKI therapy significantly improved the OS and PFS in different types of thyroid cancer (5). Nevertheless, a significant breakthrough of TKIs in ATC has not come (1). In the published prospective clinical trials, clinics have tried to detect the efficacy of TKIs in ATC patients. Several issues deserve our attention. Huan and Panayiotis’s trials were terminated due to poor accrual, showing a 6-month survival of 36% for imatinib and a median PFS of 1.9 months for sorafenib (6, 7); Keith’s trial was halted because a stopping rule was triggered, with a median PFS of 62 days and a median OS of 111 days (8); Lori’s trial was halted due to the unmet response rate threshold, and reported a median PFS of 2.6 months and a median OS of 3.2 months (9). Although Takuya’s trial finished, the final results were disappointing (1-year OS rate: 11.9%) (10).

Considering the above reasons, the data reported in prospective clinical trials might be incomplete, and the bias was made. Therefore, we are eager to detect the benefits of TKIs for patients with ATC in real-world clinical practice.

Similarly, owing to the low incidence rate of ATC, the effects of TKIs in ATC displayed in retrospective studies were also inconsistent. To comprehensively demonstrate the efficacy of TKIs in treating ATC patients, we reconstructed the patient-level survival data to show the survival curves directly and meta-analyzed the response rates in real-world retrospective studies.

## Methods

This study was conducted according to the Preferred Reporting Items for Systematic Reviews and Meta-analyses (PRISMA) guideline (11).

### Literature search

A systematic literature search was performed to identify real-world retrospective studies eligible for this analysis. The search was conducted in four online databases (PubMed, Web of Science, Embase, and Cochrane CENTRAL) on September 3, 2023.

The search terms were:

1. “anaplastic thyroid cancer or anaplastic thyroid carcinoma”.
2. “tyrosine kinase inhibitor or sorafenib or lenvatinib or regorafenib or imatinib or cabozantinib or donafenib or apatinib”.
3. “trial or study”.

### Inclusion and exclusion criteria

The inclusion criteria were as follows: (1) patients were diagnosed with ATC; (2) patients were treated with TKI-based therapy; (3) efficacy data were available (number of patients in a subgroup or overall population should be ≥ 10); (4) real-world retrospective studies were published in English. Meeting abstracts and case reports were excluded. The primary endpoints were PFS, OS, objective response rate (ORR), and disease control rate (DCR).

### Data extraction

Two authors independently extracted the detailed data of the study design, number of patients, median age, therapeutic strategies, survival outcomes, and responses. In addition, reconstructed survival data were collected from published Kaplan-Meier curves using the Scanlt software (version 2.0.8.0).

### Risk assessment

Funnel plots, sensitivity analysis, and Egger’s tests were calculated to evaluate the publication bias.

### Statistical analysis

R software (version 4.2.2) was applied to conduct the analyses. The pooled time-to-event survival data and response rates were analyzed by running the “metaSurvival” package and the “meta” package. A random-effects model was performed to reduce the heterogeneity.

## Results

**Figure 1:**
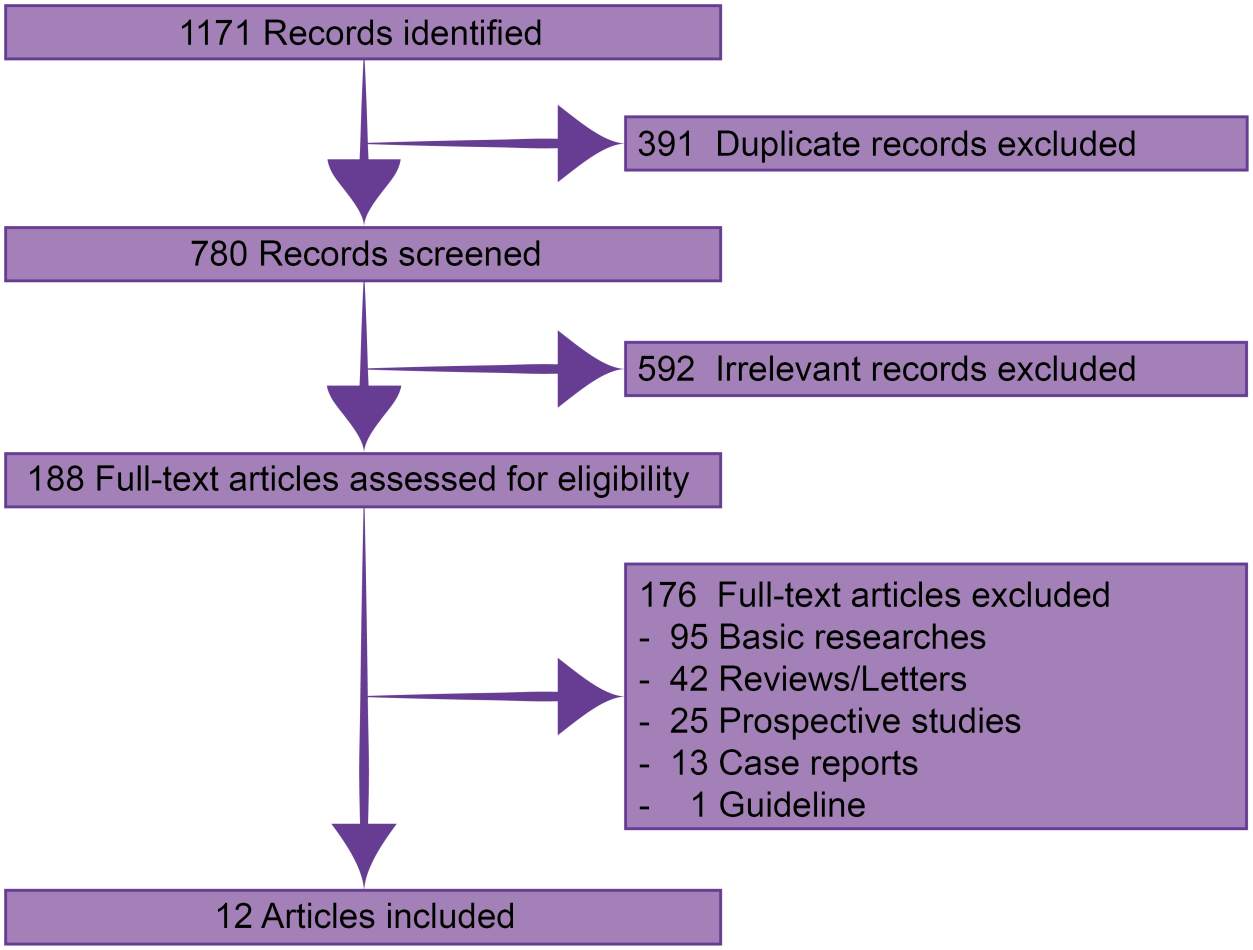
Flow diagram of the selecting process.

Through systematic searching, 1171 records were identified. After excluding 391 duplicate and 592 irrelevant records, 188 were available in full-text assessment. Finally, 12 published real-world retrospective studies with 227 patients were eligible for this analysis (**Figure 1**) (12–23).

Two of the 12 studies were multi-center studies (16, 23), while the other 10 were single-center studies. Therapeutic regimens comprised anlotinib, lenvatinib, dabrafenib plus trametinib, vemurafenib, pembrolizumab plus dabrafenib and trametinib, pembrolizumab plus lenvatinib, pembrolizumab plus trametinib, and sorafenib. The median or mean age was over 60 (**Table 1**).

**Table 1:** Basic characteristics of eligible retrospective studies.

Study	Publication year	Inclusion period	Design	No.patients	Age	Drug
Xucai Zheng (10)	2023	2017.07-2022.02	Single-center study	25	Median 65.01 (range 40-85)	Anlotinib
Hiroyuki Iwasaki (11)	2023	2011.04-2022.07	Single-center study	36	Median 72.33 (range 47-85)	Lenvatinib
Daisuke Murayama (12)	2022	2015.11-2021.05	Single-center study	26	Median 73 (IQR 65-79)	Lenvatinib
Jun Park (13)	2021	1995.11-2020.05	Single-center study	19	Mean 66.4 ± SD1.3	1. Dabrafenib plus trametinib 2. Lenvatinib 3. Vemurafenib
Mijin Kim (14)	2021	2016.08-2019.12	Multi-center study	14	Median 65.6 (IQR 59.7-72.1)	Lenvatinib
Haruhiko Yamazaki (15)	2020	2015.06-2017.08	Single-center study	12	Median 78 (range 63-89)	Lenvatinib
Hiroyuki Iwasaki (16)	2020	2011.01-2019.04	Single-center study	16	Mean 73 (range 47-89)	Lenvatinib
Soo Young Kim (17)	2020	2015.10-2018.02	Single-center study	18	Mean 64.9 (range42-86)	Lenvatinib
Hiroyuki Iwasaki (18)	2018	2015.04-2017.03	Single-center study	23	Median 77 (range 42-89)	Lenvatinib
Priyanka C. Iyer (19)	2018	2015.04-2016.05	Single-center study	16	Median 67 (range 55-82)	1. Lenvatinib 2. Dabrafenib plus trametinib
Priyanka C. Iyer (20)	2018	2016.08-2017.08	Single-center study	12	Median 60 (range 47-84)	Pembrolizumab plus 1. Dabrafenib plus trametinib 2. Lenvatinib 3. Trametinib
Yasuhiro Ito (21)	2017	2014.04-2014.09	Multi-center study	10	Median 72 (range 60-82)	Sorafenib

**Figure 2:**
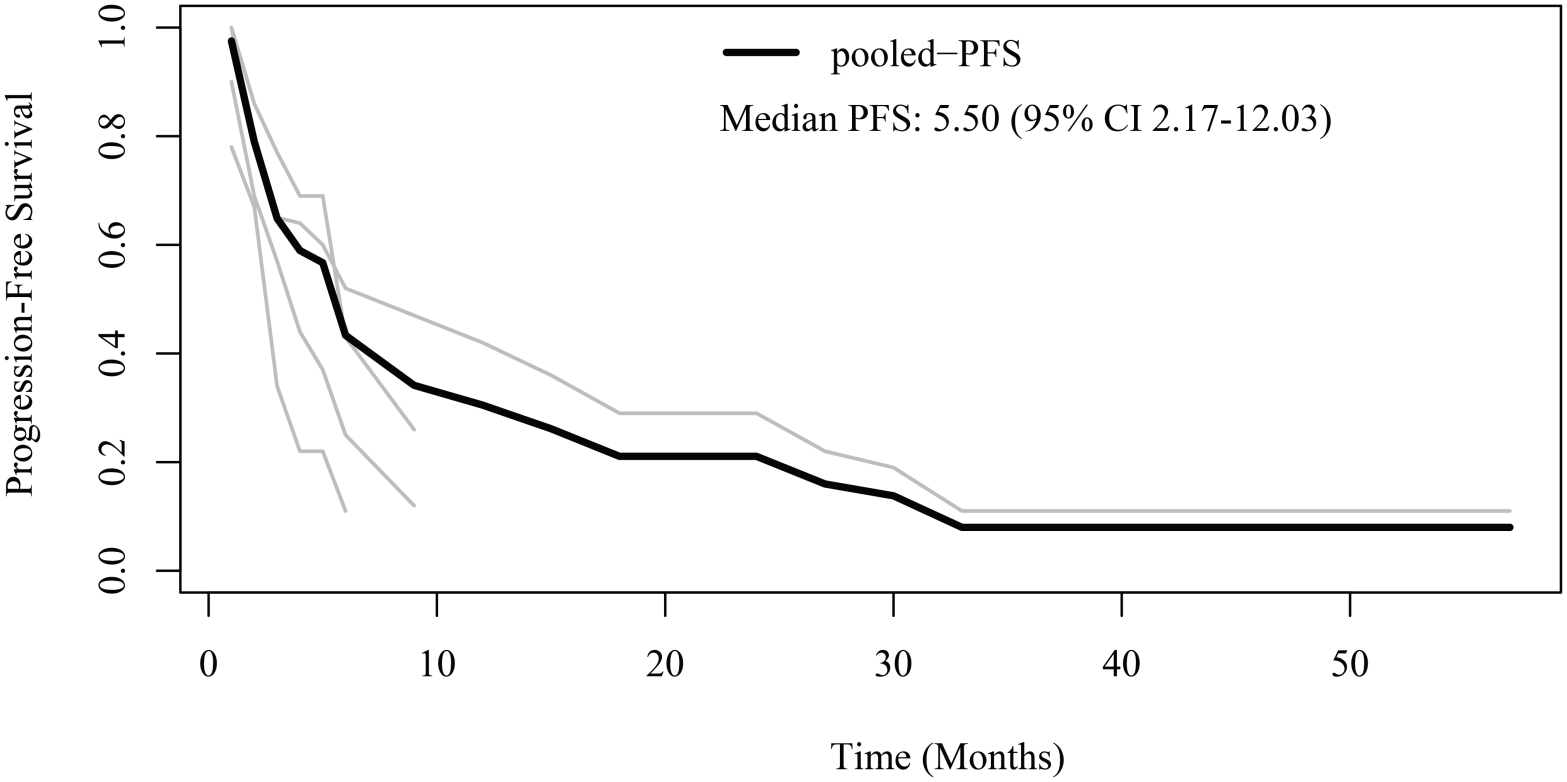
Reconstructed progression-free survival (PFS) curves of anaplastic thyroid cancer patients treated with tyrosine kinase inhibitor therapy. The dark line indicates the pooled PFS curve. The light lines are the PFS curves reconstructed from the four enrolled studies.

Regarding PFS, patient-level data were extracted from four studies (12, 16, 21, 23). The pooled median PFS was 5.5 months (95% CI 2.17-12.03) (**Figure 2**). The 6-month, 1-year, 2-year, and 3-year PFS rates were 43.3%, 30.5%, 21.1%, and 8.0%, respectively.

**Figure 3:**
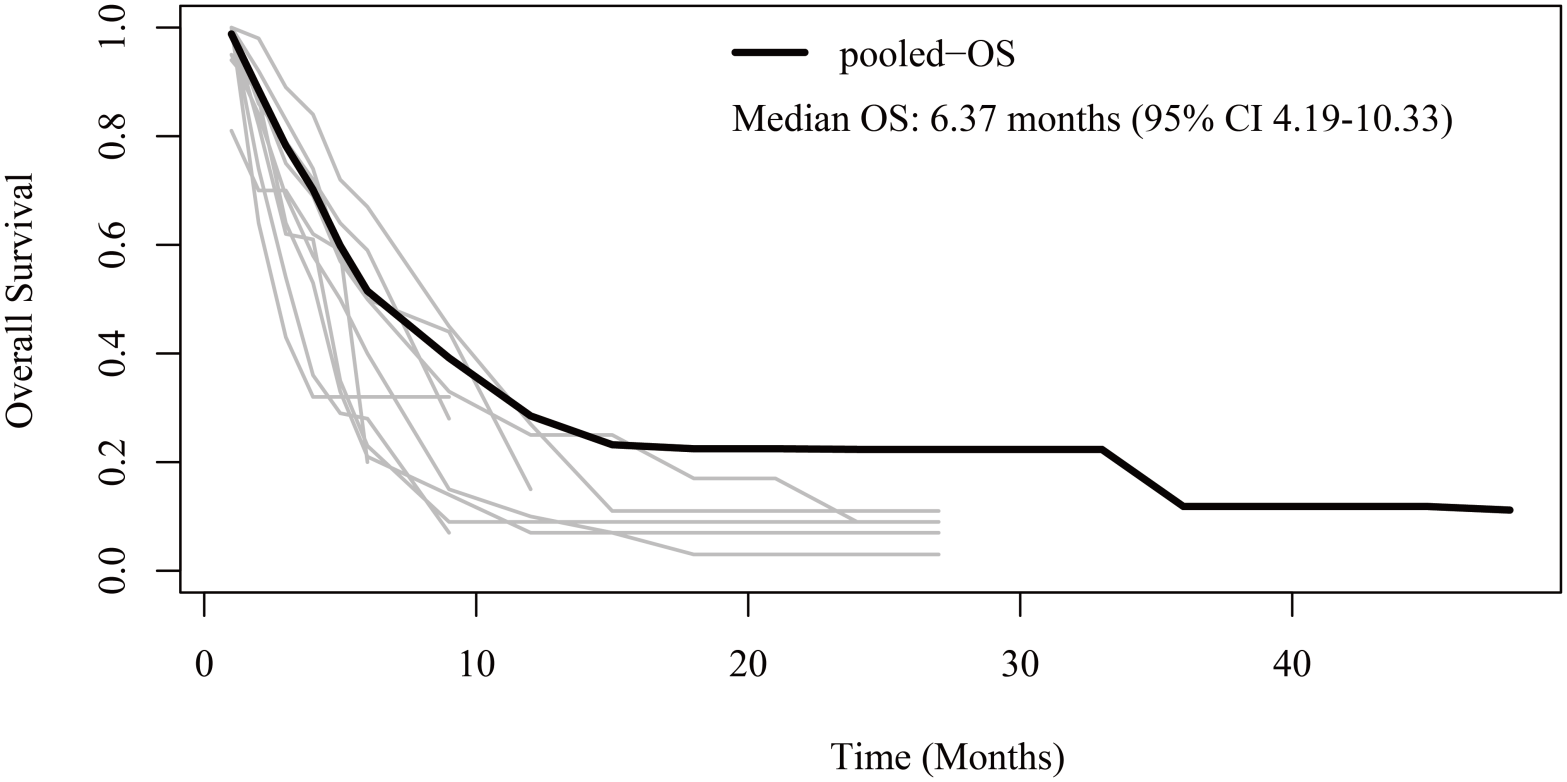
Reconstructed overall survival (OS) curves of anaplastic thyroid cancer patients treated with tyrosine kinase inhibitor therapy. The dark line indicates the pooled OS curve. The light lines are the OS curves reconstructed from the ten enrolled studies.

Regarding OS, patient-level data were reconstructed from 10 studies (13–19, 21–23). The pooled OS was 6.37 months (95% CI 4.19-10.33) (**Figure 3**). The 6-month, 1-year, 2-year, and 3-year OS rates were 51.5%, 28.5%, 22.3%, and 11.8%, respectively.

**Figure 4:**
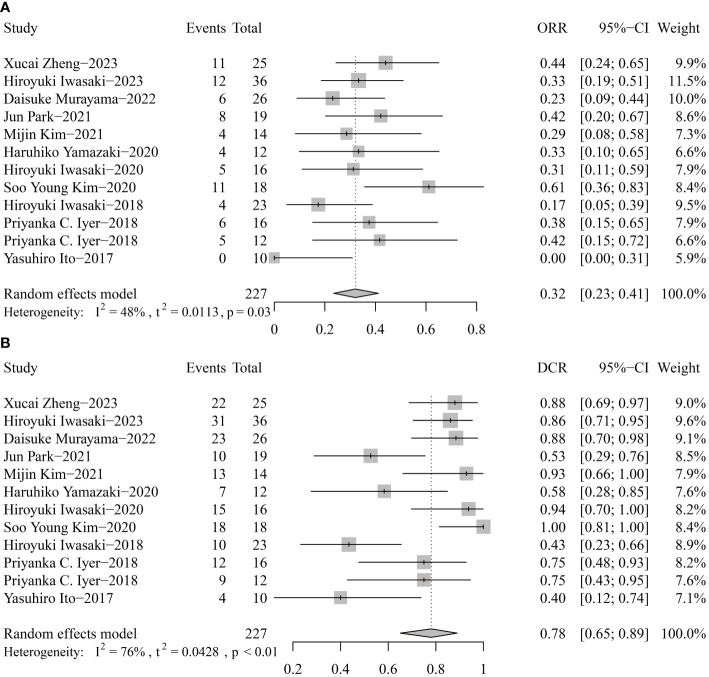
Pooled objective response rate (ORR, **A**) and disease control rate (DCR, **B**) of tyrosine kinase inhibitor therapy in anaplastic thyroid cancer patients.

For response rates, the overall ORR and DCR were 32% (95% CI 23-41) and 40% (95% CI 65-89) (**Figure 4**). Publication bias was not found according to the funnel plots, sensitivity analyses, and Egger’s tests.

## Discussion

In this analysis based on real-world clinical practice, we found that the median PFS was 5.50 months, the median OS was 6.37 months, the ORR was 32%, and the DCR was 40% in ATC patients treated with TKI-based therapy. Our results indicated that TKI treatment could be an effective therapy for ATC patients.

Chemotherapy has been playing a critical role in the treatment for ATC. Comparing TKI treatment with chemotherapy in ATC patients can be an interesting topic. In 2014, Julie A Sosa published a clinical trial and the data showed that the median OS was 4.0 months when ATC patients received paclitaxel and carboplatin (24). Another prospective study reported by Naoyoshi Onoda found that weekly paclitaxel administration in ATC patients showed a median OS of 6.7 months, with a 21% ORR (25). Moreover, in K B Ain’s trial, a total response rate of 53% was achieved when patients were treated with 96-hour continuous infusion paclitaxel (26). Accordingly, the survival time of patients enrolled in our study was not superior to that of patients in the above trials.

Although our sensitivity analyses did not find any heterogeneity, patients in 10 of the 12 studies received lenvatinib-based therapy. In Priyanka C. lyer’s study, ATC patients treated with dabrafenib plus trametinib showed a shorter median OS compared with patients treated with lenvatinib (7.4 months vs. 10.4 months) (21). For patients with BRAF V600E-mutant, dabrafenib plus trametinib treatment achieved a median OS of 14.5 months and a median PFS of 6.7 months (27). Therefore, confirming the accurate population of ATC patients who could benefit more from lenvatinib, dabrafenib plus trametinib, or other targeted therapy may become one of the future tasks.

By comparing our results with the data in prospective clinical trials, we noticed that the survival time reported in retrospective studies was longer than in prospective studies. In Panayiotis S’s trial, patients were treated with sorafenib, and the median OS and PFS were 3.9 months and 1.9 months (6). In another trial reported by Lori W, the median OS and PFS of patients who received lenvatinib were 3.2 months and 2.6 months (9). We deduced that one of the most important reasons was that patients enrolled in prospective trials had been heavily treated with previous systemic therapy. In contrast, most patients collected in retrospective studies received targeted therapy as their first-line treatment. However, not all data reported in the prospective studies were disappointing. For example, in Shunji Takahashi’s phase II study, ATC patients who received lenvatinib had a response rate of 24% and a median PFS of 7.4 months, revealing that only one patient experienced a treatment-related toxicity leading to discontinuation (28).

Immunotherapy has revolutionized the treatment of malignant tumors. In patients with ATC, anti-PD-L1 therapy showed positive results in the subset of patients with PD-L1 ≥ 50%, with a response rate of 35% (29). Combining anti-PD-L1 and anti-angiogenic targeted therapy has significantly improved the survival outcomes in cancer patients. For example, hepatocellular carcinoma, gastric cancer, and non-small cell lung cancer (30–32). Whether targeted therapy in combination with immunotherapy plays a novel efficacy in advanced ATC needs more future explorations.

In terms of treatment-related adverse events, most of the retrospective studies failed to report a comprehensive safety profile. For grade ≥ 3 adverse events, the highest incidence of TKI were hyponatremia (13% for dabrafenib and trametinib) (21), fatigue (20% for lenvatinib) (22), and hypertension (20% for lenvatinib) (23). However, we may not be sure whether the adverse events recorded are accurate because of the long inclusion period in retrospective studies. Accordingly, we reviewed the adverse events of TKIs reported in the prospective studies. The most incidence of grade ≥ 3 adverse events were loss of appetite for lenvatinib (16%) (10), hypertension for lenvatinib and pazopanib (> 20%) (8, 9, 33), rash for sorafenib (15%) (6), and lymphopenia for imatinib (45%) (7). Additionally, the tolerability of TKIs is also essential. For pazopanib, the discontinuation was mainly attributed to disease progression and treatment-related adverse events (hemorrhage, hypertension, and radiation recall tracheitis) (8). For lenvatinib, ATC patients discontinued due to treatment-related adverse events (9, 10, 33). Therefore, caution of treatment-related adverse events and tolerability of TKIs in ATC patients are warranted.

There were several limitations in this study. The baseline characteristics of the patients in each study were various. Because some patients might have received surgery, radiotherapy, or other therapeutic strategies, these elements could partially impact the final survival outcomes. In addition, owing to the long accrual time, detailed data on each patient, such as adverse events and drug discontinuation, might be incomplete. With the development of medical science, novel therapeutic strategies would contribute to improving survival time.

## Conclusion

TKI therapy could be an effective therapeutic option for patients with ATC. However, there is a long way to go to prolong the survival time further and break the bottleneck.

## Data availability statement

The raw data supporting the conclusions of this article will be made available by the authors, without undue reservation.

## Author contributions

BK: Data curation, Investigation, Writing – original draft, Writing – review & editing. WZ: Writing – original draft, Writing – review & editing. GL: Writing – original draft, Writing – review & editing. CF: Writing – original draft, Writing – review & editing. RC: Data curation, Writing – original draft, Writing – review & editing. BW: Conceptualization, Data curation, Formal Analysis, Funding acquisition, Investigation, Methodology, Project administration, Resources, Software, Supervision, Validation, Visualization, Writing – original draft, Writing – review & editing.
